# Celecoxib analogues disrupt Akt signaling, which is commonly activated in primary breast tumours

**DOI:** 10.1186/bcr1294

**Published:** 2005-08-01

**Authors:** Jill E Kucab, Cathy Lee, Ching-Shih Chen, Jiuxiang Zhu, C Blake Gilks, Maggie Cheang, David Huntsman, Erika Yorida, Joanne Emerman, Michael Pollak, Sandra E Dunn

**Affiliations:** 1 British Columbia Research Institute for Children's and Women's Health, Department of Pediatrics, University of British Columbia, Vancouver, British Columbia, Canada; 2 Division of Medical Chemistry and Pharmacognosy, The Ohio State University, Columbus, Ohio, USA; 3 Genetic Pathology Evaluation Centre, Vancouver Hospital and Health Sciences Centre and BC Cancer Agency, Vancouver, British Columbia, Canada; 4 Department of Anatomy, Faculty of Medicine, University of British Columbia, Vancouver, British Columbia, Canada; 5 Division of Experimental Medicine, Department of Medicine and Department of Oncology, McGill University, Montreal, Quebec

## Abstract

**Introduction:**

Phosphorylated Akt (P-Akt) is an attractive molecular target because it contributes to the development of breast cancer and confers resistance to conventional therapies. Akt also serves as a signalling intermediate for receptors such as human epidermal growth factor receptor (HER)-2, which is overexpressed in 30% of breast cancers; therefore, inhibitors to this pathway are being sought. New celecoxib analogues reportedly inhibit P-Akt in prostate cancer cells. We therefore examined the potential of these compounds in the treatment of breast cancer. The analogues were characterized in MDA-MB-453 cells because they overexpress HER-2 and have very high levels of P-Akt.

**Methods:**

To evaluate the effect of the celecoxib analogues, immunoblotting was used to identify changes in the phosphorylation of Akt and its downstream substrates glycogen synthase kinase (GSK) and 4E binding protein (4EBP-1). *In vitro *kinase assays were then used to assess the effect of the drugs on Akt activity. Cell death was evaluated by poly(ADP-ribose) polymerase cleavage, nucleosomal fragmentation and MTS assays. Finally, tumour tissue microarrays were screened for P-Akt and HER-2 expression.

**Results:**

OSU-03012 and OSU-O3013 inhibited P-Akt and its downstream signalling through 4EBP-1 and GSK at concentrations well below that of celecoxib. Disruption of P-Akt was followed by induction of apoptosis and more than 90% cell death. We also noted that the cytotoxicity of the celecoxib analogues was not significantly affected by serum. In contrast, the presence of 5% serum protected cells from celecoxib induced death. Thus, the structural modification of the celecoxib analogues increased P-Akt inhibition and enhanced the bioavailability of the drugs *in vitro*. To assess how many patients may potentially benefit from such drugs we screened tumour tissue microarrays. P-Akt was highly activated in 58% (225/390) of cases, whereas it was only similarly expressed in 35% (9/26) of normal breast tissues. Furthermore, HER-2 positive tumours expressed high levels of P-Akt (*P *< 0.01), supporting *in vitro *signal transduction.

**Conclusion:**

We determined that Celecoxib analogues are potent inhibitors of P-Akt signalling and kill breast cancer cells that overexpress HER-2. We also defined an association between HER-2 and P-Akt in primary breast tissues, suggesting that these inhibitors may benefit patients in need of new treatment options.

## Introduction

Receptor tyrosine kinases (RTKs) are commonly overexpressed in breast cancer, in which they promote tumour growth and metastasis. For example, insulin-like growth factor (IGF)-1 receptor is an RTK that is overexpressed in about 70% of breast cancers [1,2]. It is fundamentally linked to malignant transformation *in vitro *and *in vivo *[3]. IGF-1 receptor is also important for breast cancer invasion and metastasis [4]. Human epidermal growth factor receptor (HER)-2 is yet another important RTK that is overexpressed in 25–30% of invasive ductal breast carcinomas and is associated with poor patient prognosis and increased risk for recurrence [5]. Transgenic mouse models show that HER-2 promotes the development of mammary tumours [6]. Armed with this knowledge, it would appear that finding a convergence point between IGF-1 receptor and HER-2 would provide a new way to target treatment.

A common feature of IGF-1 receptor and HER-2 is signalling through the phosphatidylinositol 3-kinase (PI3K)/Akt pathway [7]. These RTKs activate PI3K, which then catalyzes the production of lipid molecules, including phosphatidylinositol-3,4,5-triphosphate [8]. The phosphatidylinositol-3,4,5-triphosphate lipids trigger the attachment of Akt to the plasma membrane, where it subsequently becomes phosphorylated at two key sites, threonine 308 and serine 473, resulting in its full activation. Threonine 308 is phosphorylated by phosphoinositide-dependent kinase (PDK)-1, whereas the mechanism of phosphorylation at serine 473 is a little more controversial. There are several theories to explain serine 473 phosphorylation, including the action of integrin-linked kinase, autophosphorylation, or an as yet unidentified PDK-2 [9]. Once Akt is fully activated it dissociates from the plasma membrane and proceeds to phosphorylate both cytoplasmic and nuclear target proteins, notably glycogen synthase kinase (GSK)-3β [10], p27^Kip ^[11], mammalian target of rapamycin [12] and forkhead transcription factors [13]. The diverse targets of phosphorylated Akt (P-Akt) regulate proliferation, invasion and evasion of apoptosis. Thus, Akt is a major convergence point for RTK signalling in breast cancer, and so inhibiting it could provide a new therapeutic avenue.

Akt has become a favoured second messenger from a therapeutic standpoint because numerous studies point toward it as a central molecule in the development of cancer. Evidence from experimental models suggests that Akt is a key regulator of tumour development and progression. There are three isoforms of Akt (Akt1, Akt2 and Akt3), which exhibit 80% amino acid sequence homology. The overexpression of each of these isoforms leads to malignant transformation (for review, see Hill and Hemmings [14]).

Transgenic mouse models have been instrumental in addressing the role of Akt in mammary tumour development. For example, mammary tumours that develop from HER-2 transgenic mice clearly overexpress P-Akt [15]. One then questions whether P-Akt truly facilitates the development of the tumours. Hutchinson and coworkers [15] addressed this by engineering bitransgenic mice expressing both HER-2 and constitutively activated Akt1 in the mammary gland. When Akt1 was constitutively expressed the mice developed tumours at a much faster rate than in those that only expressed HER-2. Thus, activated Akt1 plays a functional role in promoting the development of HER-2 positive mammary tumours. Importantly, analysis of primary tumour tissues shows that Akt1 is frequently expressed and highly activated in patients [16]. Akt1 kinase activity is significantly increased in approximately 40–50% of tumour samples from patients with breast (19/50 cases), ovary (11/28 cases) and prostate cancer (16/30 cases) relative to normal tissue [16]. In addition to its role in cancer development, Akt also promotes the survival of tumour cells when confronted with chemotherapeutics and radiation. For example, in breast cancer cells expression of constitutively active Akt1 reduced the ability of doxorubicin [17] or ionizing radiation [18] to induce apoptosis. On the other hand, the PI3K inhibitor Ly294002 or a dominant-negative Akt1 sensitized cancer cells to chemotherapy [17]. These data suggest that inhibiting P-Akt signalling in tumours could have important therapeutic applications.

There is intense interest in targeting RTKs and signal transduction intermediates such as Akt for the treatment of cancer [19]. One approach to inhibiting P-Akt is to target upstream activators of this pathway. For example, patients with tumours expressing HER-2 can be treated with herceptin, a monoclonal antibody that blocks the activation of the receptor [20] and subsequently inhibits Akt phosphorylation [21]. However, fewer than 30% of patients treated with herceptin initially respond [22]. Within that population of patients who initially respond to herceptin, some subsequently develop resistance [22]. A second approach is to inhibit Akt directly. However, no Akt inhibitors are available to patients, although the proof of principle was elegantly provided in a study using an Akt dominant negative (Akt-DN) inhibitor [23]. Targeted disruption of Akt by Akt-DN inhibited the growth of the breast cancer cell lines ZR75-1 and MDA-MB-453 *in vitro*. The Akt-DN also caused the cells to undergo apoptosis. Adenoviral mediated delivery of Akt-DN also had an impressive antitumour effect *in vivo*. That study provided the first evidence that targeted disruption of Akt induces apoptosis and suppresses tumour formation in mice. Thus, there is growing interest in the discovery of Akt inhibitors. Potential future drug candidates include phosphatidylinositol analogues that bind specifically to the PH domain of Akt and have been shown to inhibit its phosphorylation in cancer cells [24]. A novel Akt inhibitor was also recently identified from the National Cancer Institute Diversity Set, and preclinical evidence [25] is promising. The inhibitor termed API-2 is a tricyclic nucleoside that selectively kills cancer cells that express high levels of activated Akt [25].

One of the newest classes of Akt inhibitors to be developed are those derived from the common anti-inflammatory drug celecoxib. Initially, it was thought that celecoxib would be a good inhibitor of Akt [26]. However, it was realized that the celecoxib concentrations achievable in patients were of the order of 3 μmol/l [27], whereas 50 μmol/l or greater is required to inhibit Akt activation in prostate [28] and breast cancer cells (Kucab and coworkers, unpublished data). Nonetheless, the observation that celecoxib inhibited P-Akt set the course for the development of analogues that optimally disrupt this pathway at lower concentrations. New celecoxib analogues have since been developed that are superior inhibitors of Akt phosphorylation [29]. The compounds referred to as OSU03012 and OSU03013 inhibited PDK-1 kinase activity *in vitro *(50% inhibitory concentration 2–5 μmol/l) and prevented Akt phosphorylation in prostate cancer cells at 1–10 μmol/l. Upon longer exposure, these inhibitors induce apoptosis in PC-3 cells. As a part of the Rapid Access to Preventive Intervention Development (RAPID) programme at the US National Cancer Institute [30], a panel of 60 cancer cells lines were screened for response to OSU03012 and OSU03013. It was determined that the compounds were potent inhibitors of tumour cell growth, with an average 50% inhibitory concentration of about 1–2 μmol/l [29]. OSU03012 has also been given orally at a dose of 200 mg/kg for 1 month without overt signs of toxicity as part of the RAPID program (Chen and coworkers, unpublished data). Characterization of the celecoxib analogues thus far indicates that they could be very useful for safely treating many types of cancer.

We therefore further explored the promise of these celecoxib analogues for the treatment of breast cancer. An extensive study of these analogues has not previously been performed in models of breast cancer. We were also curious as to whether serum proteins attenuated the cytotoxic effect of the new analogues as they do with celecoxib [31]. The compounds were evaluated in a HER-2 overexpressing breast cancer cell line, namely MDA-MB-453, which is well characterized for having very high levels of P-Akt. We report herein that both of the celecoxib analogues inhibited Akt phosphorylation and Akt kinase activity. The compounds also inhibited phosphorylation of substrates downstream of Akt (GSK and 4EBP-1). Furthermore, OSU-03012 and OSU-03013 initiated the apoptotic pathway, resulting in under 90% cell viability within 24 hours. We then addressed how often Akt is activated in primary tumours to estimate the number of patients that might benefit from these small molecule inhibitors. We determined that P-Akt was moderately to highly expressed in 58% of primary tumours, suggesting that these inhibitors could potentially be used to treat a substantial number of patients. Furthermore, we found that Akt was commonly activated in tumours that overexpress HER-2. These data therefore provide evidence to support further preclinical development of celecoxib analogues for the treatment of breast cancer, particularly in cases in which HER-2 is overexpressed.

## Materials and methods

### Effect of celecoxib analogues on Akt signalling and apoptosis

The breast cancer cell lines (MDA-MB-453, MCF-7, T47D, MDA-MB-231 and HBL100) were obtained from the American Tissue Culture Collection (Manassas, VA, USA). The 184htrt cells were a gift from Dr J Carl Barrett (National Cancer Institute, Bethesda, MA, USA). All of the experiments were performed in the presence of 5% foetal calf serum, RPMI-1640, with the exception of the 184htrt cells, which were grown as previously described [32]. The celecoxib analogues were synthesized as previously described by us [29]. Ly294002 was purchased from Sigma (St. Louis, MO, USA) and celecoxib was obtained from Pharmacia (St Louise, MO, USA). All compounds were dissolved in dimethyl sulphoxide.

To study the effect of the inhibitors on signal transduction, the cells were treated with either the PI3K/Akt inhibitor Ly294002 (30 μmol/l), or OSU03012 or OSU03013 inhibitor, each at 5 and 10 μmol/l for 2 hours or at later times points, as indicated. Comparisons were also made to the parent compound, celecoxib, at concentrations of 50 μmol/l and 75 μmol/l. Whole cell extracts were prepared in accordance with the protocol for P-Akt detection by Cell Signaling Technologies (CST Beverly, MA, USA). All antibodies were purchases from CST unless otherwise indicated. P-AKT^ser473^, P-Akt^thr308^, total AKT, P-4EBP-1, P-S6, P-Erk, total Erk, P-MK2, MK2, P-GSK (Upstate Biotechnology Inc, Lake Placid, NY, USA) and actin (Santa Cruz Biotechnology, CA, USA) were detected by analyzing 50 μg total protein separated on a 12% acrylamide gel.

Akt kinase activity was assessed using a modified assay. Nonradioactive Akt kinase assays were performed using a protocol modified from that of Cell Signaling Technologies. In brief, 500 μg protein, from cells treated as described above, were immunoprecipitated with the 5G3 pan-Akt antibody (CST) overnight at 4°C. The following day the Akt–antibody complexes were incubated with protein G coated agarose beads. The immunoprecipitated complexes were washed and then incubated for 30 minutes at 30°C in kinase buffer with 1 μg of recombinant GSK-3 protein (CST) and 200 μmol/l ATP. To stop the reaction, 15 μl of 4× SDS sample buffer with β-mercaptoethanol was added. The assays were boiled for 5 min and then one-third of each reaction was separated on a 12% acrylamide gel and immunoblotted. The blots were analyzed using antibodies to P-GSK-3 protein, as well as total recombinant GSK-3 and total Akt.

In order to assess the effect of the compounds on apoptosis, the MDA-MB-453 cells were treated with Ly294002, celecoxib, or the analogues at the indicated concentrations for 12 or 24 hours and poly(ADP-ribose) polymerase (CST) cleavage was examined in the treated cells by immunoblotting. Cell extracts were also subjected to the Cell Death Detection Assay according to the manufacturer's instructions (Roche Diagnostics, Laval, QC, Canada). The viability of MDA-MB-453 was determined 24 hours after exposure to celecoxib, the analogues, or Ly294002 using the Celltiter 96 Aqueous cell proliferation/survival assay (Promega, Madison WI, USA), as previously described [33]. The influence of serum proteins was also examined. The MDA-MB-453 cells were treated with the test compounds as indicated in either 5% foetal bovine serum/RPMI or 0.1% foetal bovine serum/RPMI, and cytotoxicity was evaluated 24 hours later using the MTT assay.

### Examination of phosphorylated Akt in primary breast tissues

For construction of the tumour tissue microarray (TMA), 481 primary breast cancer samples were obtained from archival cases at Vancouver General Hospital dating between 1974 and 1995. Patient information and tumour pathology are summarized in Additional file 1. Anonymous coding was used to protect patient rights and the samples were procured in accordance with the guidelines established by the Vancouver General Hospital. Tumour samples were taken before initiation of cancer treatment, and were formalin fixed and embedded in paraffin. The TMA was constructed as previously described by us [1].

For detection of P-Akt, the tissues underwent antigen retrieval by incubating the slides for 30 min in 10 mmol/l citrate buffer (pH 6.0) at 60–90°C in a vegetable steamer. Endogenous peroxidases were quenched by incubating the sections for 10 min in 3% H_2_O_2_. Additionally, nonspecific interactions were blocked for 30 min using a non-serum-blocking reagent (DAKO, Denmark), followed by 20 min with an avidin/biotin blocking solution (DAKO, Carpenteria, Ca, USA). The primary antibody (Phospho-AKT^S473 ^IHC Specific; CST) was diluted 1:250 with a 1% bovine serum albumin solution, applied to the sides and incubated overnight at 4°C. For signal amplification, we then used the LSAB+ System (DAKO), which involved incubation with a biotinylated secondary antibody followed by streptavidin treatment. P-Akt was visualized by addition of NovaRed substrate (Vector Laboratories, Burlingame, CA, USA) and the sections were counter-stained with haematoxylin. Additionally, a negative control reaction with no primary antibody was performed for each slide in parallel.

The scoring system for P-Akt expression was as follows: 0 = negative, 1 = weak, 2 = moderate and 3 = high staining intensity. Of the 481 cases on the array, 438 contained invasive carcinoma and the characteristics of these patients are described in Table 1. A total of 390 invasive carcinoma cases were interpretable for P-Akt expression. In most cases, P-Akt expression was detected primarily in the cytoplasm, although nuclear staining was occasionally observed. The majority of P-Akt was predominantly expressed in the tumour epithelial cells, although there was also notable staining of the endothelial cells. P-Akt was not expressed in the stroma. To assess P-Akt staining in normal breast tissue, 26 samples were obtained from patients who underwent reduction mammoplasties at Vancouver General Hospital from 2000 to 2001. The tissues were formalin fixed and paraffin embedded. One section of each sample was stained with haematoxylin and eosin and then assessed by a pathologist to ensure presence of normal breast epithelium. P-Akt immunohistochemistry was performed on the normal tissues as described above for the TMAs. Raw scores for P-Akt expression were entered into a standardized electronic spreadsheet (Excel for Windows, Microsoft, Redmond, WA, USA), processed using Deconvoluter software designed for management of TMA data, and then analyzed using the SPSS for Windows statistical software package (SPSS version 11; SPSS, Chicago, IL). The difference in P-Akt expression between normal and tumour breast tissue was calculated using χ^2 ^analysis. HER-2 R was stained with an antibody designated A485 (Dako) at a dilution of 1:500 and detected using the LSAB+ System.

## Results

### Celecoxib analogues disrupt Akt signalling in breast cancer cells and induce apoptosis

**Figure 1:**
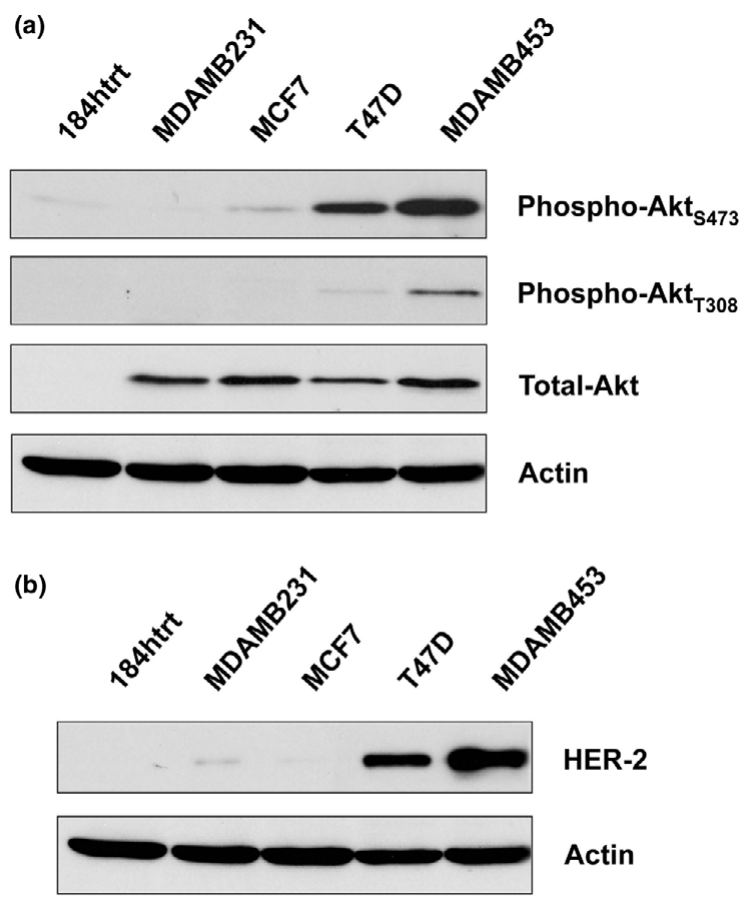
Examination of P-Akt and HER-2 levels in a panel of breast cancer cell lines. **(a) **Proteins were isolated from cells growing in log phase and the levels of phosphorylated Akt (P-Akt) were then assessed using antibodies to serine 473 and threonine 308. MDA-MB-453 cells expressed the highest levels of activated Akt relative to the other lines. There were no differences in the levels of total Akt with the exception of the preneoplastic cell line 184htrt. Actin was detected as a loading control. **(b) **Human epidermal growth factor receptor (HER)-2 protein expression was evaluated in a panel of breast cell lines. The MDA-MB-453 and T47D cells expressed HER-2 whereas the other cell lines did not.

We screened a panel of breast cell lines for P-Akt to find the one with the highest levels. Our panel included the preneoplastic cell line 184htrt and the cancer cell lines T47D, MCF-7, MDA-MB-231 and MDA-MB-453. The MDA-MB-453 cells expressed the highest level of P-Akt, and therefore they were extensively used to characterize the effect of the celecoxib analogues on the Akt pathway (Fig. 1a). This cell line also expressed the highest level of HER-2 (Fig. 1b).

**Figure 2:**
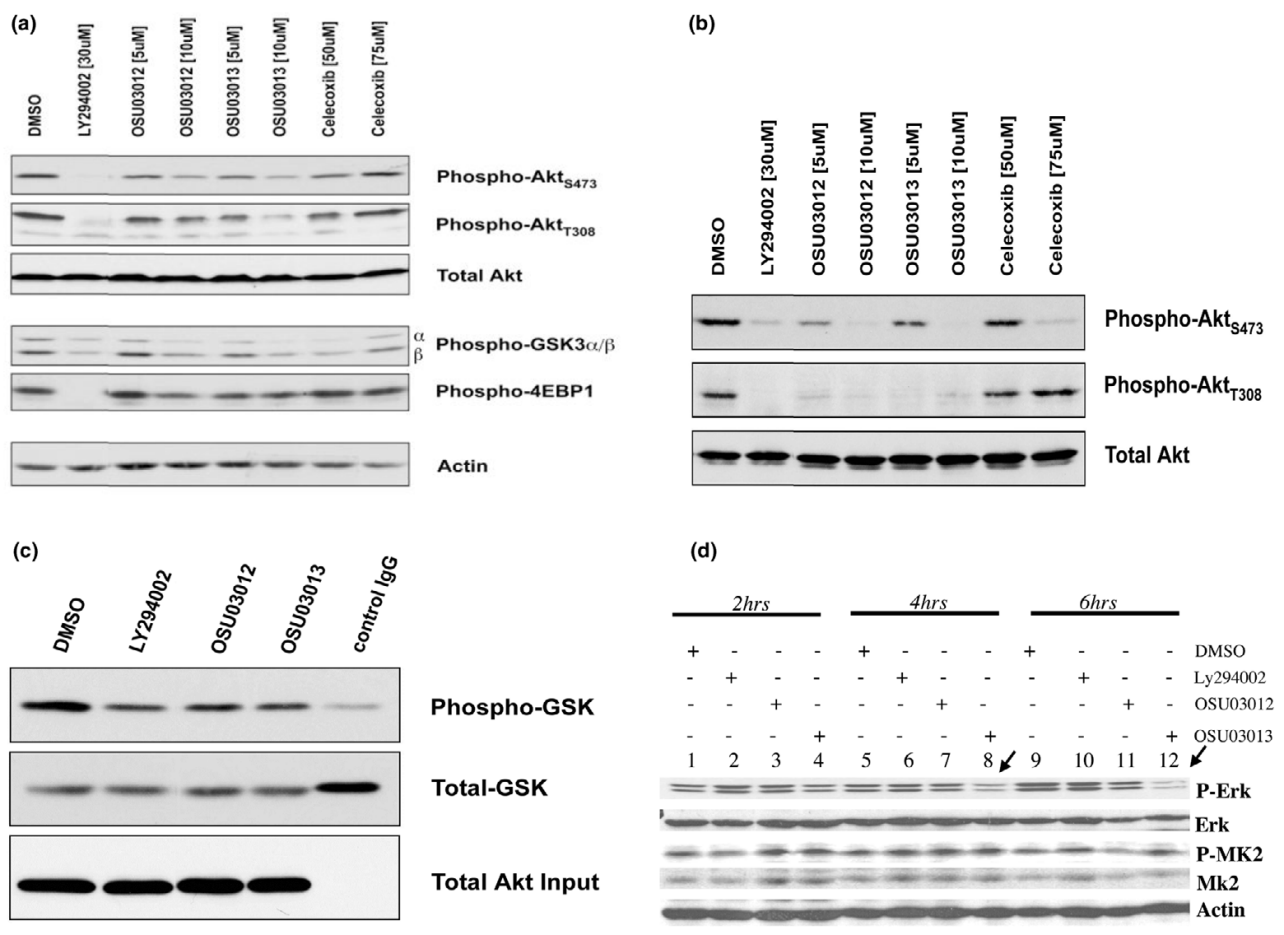
Impact of signal transduction inhibitors on Akt signalling. **(a) **The MDA-MB-453 cells were treated for 2 hours with Ly294002 (30 μmol/l), OSU03012, OSU03013 (5 or 10 μmol/l), or celecoxib (50 or 75 μmol/l). The Celecoxib analogues inhibited Akt phosphorylation at both threonine 308 and serine 473. Phosphorylation of the Akt substrates glycogen synthase kinase (GSK) and 4E binding protein (4EBP)-1 was subsequently attenuated. In contrast, celecoxib did not have an inhibitory effect. Ly294002 inhibited signal transduction through Akt, as expected. Total Akt and actin were unaffected by exposure to the signalling inhibitors. **(b) **T47D cells were also exposed to the drugs as described above and evaluated for phosphorylated Akt (P-Akt) using antibodies to Ser473 and Thr308. Total Akt was included as a control for loading. **(c) **Cells were treated as above and Akt kinase was measured against the substrate GSK. Each of the celecoxib analogues inhibited Akt kinase activity. The degree of inhibition was similar to that with Ly294002. There was minimal nonspecific kinase activity in the absence of Akt based on the IgG control. Total Akt was evaluated as a loading control to confirm that the loss of activity was not due to differences in experimental conditions. **(d) **The MDA-MB-453 cells were treated for 2, 4, or 6 hours with dimethyl sulphoxide (DMSO), Ly294002 (30 μmol/l), OSU03012 (10 μmol/l), or OSU03013 (10 μmol/l) and probed for P-Erk1/2, total Erk, P-MK2, total MK2 and actin.

To examine the potential effect of the inhibitors, the MDA-MB-453 cells were treated for 2 hours with DMSO, Ly294002 (30 μmol/l), OSU-03012 (5 or 10 μmol/l) or OSU-03013 (5 or 10 μmol/l) and compared with celecoxib treated cells (50 or 75 μmol/l). The celecoxib analogues suppressed phosphorylation of Akt at threonine 308 and serine 473, whereas celecoxib did not (Fig. 2a). These data indicated that Akt kinase activity should be inhibited by the celecoxib analogues. In support of this, we determined that the analogues inhibited phosphorylation of the Akt substrates GSK-3β and 4E-BP1 (Fig. 2a). Similarly, the celecoxib analogues inhibited P-Akt in the T47D cells (Fig. 2b). Kinase assays were then performed on the MDA-MB-453 cells to provide direct evidence that Akt activity was lost. Each of the celecoxib analogues markedly suppressed Akt kinase activity, similar to that with the PI3K/Akt inhibitor Ly294002 (Fig. 2c).

The off-target effects of the inhibitors were then examined by focusing on the mitogen-activated protein kinase (MAPK) and p38 pathways. OSU03012 did not inhibit signal transduction through the MAPK pathway, based on a lack of P-Erk inhibition (Fig. 2d). Similar results were found for Ly294002. In contrast, OSU03013 inhibited P-Erk at 4 hours (Fig. 2d, lane 8) and more so at 6 hours (Fig. 2d, lane 12). This was an important finding because it indicated that OSU03012 specifically inhibited the Akt pathway. OSU03013 on the other hand inhibited both the Akt and MAPK pathways at the later time points. To complete this portion of the study, the potential effect of the compounds was examined in the context of the p38 pathway by monitoring P-MAPK activated protein kinase-2. The latter was not inhibited by OSU03012, OSU03013, or Ly294002 (Fig. 2d). Thus, the celecoxib analogues are potent inhibitors of the Akt pathway in HER-2 over-expressing breast cancer cells; however, OSU03012 was more specific than OSU03013.

**Figure 3:**
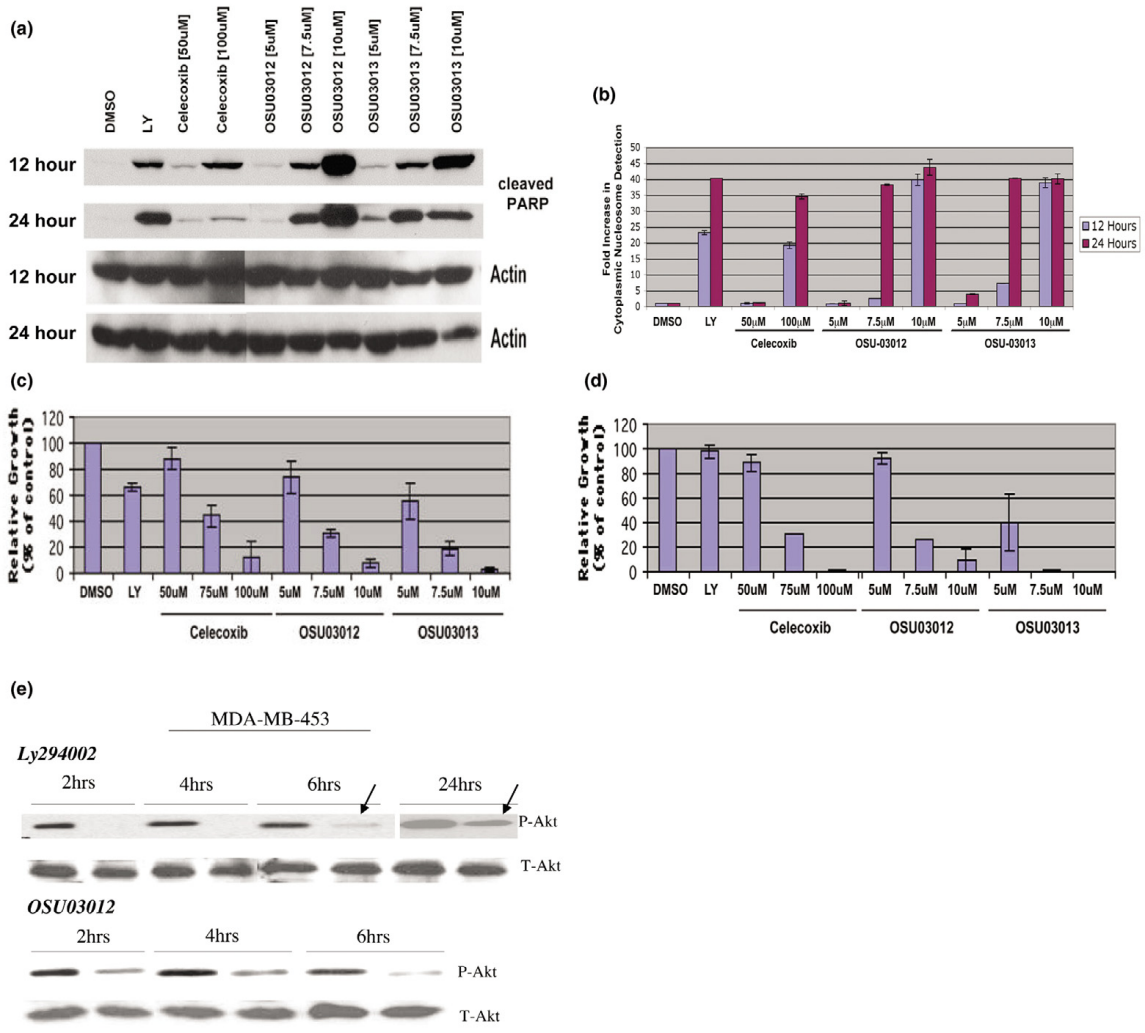
Effect of the Celecoxib analogues on apoptosis induction. To follow the fate of the cells upon Akt inhibition, indicators of apoptosis were temporally measured. Poly(ADP-ribose) polymerase (PARP) cleavage and nucleosomal fragmentation were measured after 12 and 24 hours. The cells were treated with Ly294002 (30 μmol/l), Celecoxib (50 or 100 μmol/l), OSU03012 (5, 7.5, or 10 μmol/l), or OSU03013 (5, 7.5, or 10 μmol/l) and then the cell pellets were split for PARP and nucleosomal cleavage. **(a) **There was a dose dependent increase in PARP cleavage on treatment with OSU03012 and OSU03013 at both 12 and 24 hours. LY294002 similarly induced PARP cleavage but to a lesser extent. Celecoxib at 50 μmol/l did not have sustained effects on PARP cleavage, whereas the high dose of celecoxib (100 μmol/l) did. **(b) **The induction of apoptosis was secondarily analyzed by nucleosomal fragmentation. There was a dose dependent increase in nucleosomal fragmentation upon treatment with increasing concentration of either OSU03012 or OSU03013. Likewise, Ly294002 induced fragmentation of the nucleosomes at both time points. Celecoxib at 50 μmol/l did not have such an effect. In contrast, high dose celecoxib did stimulate apoptosis. Each treatment was conducted in replicates of six on two difference occasions. **(c) **Impact of signal transduction inhibitors on cell survival. The MDA-MB-453 cells were exposed to Ly294002 (30 μmol/l), celecoxib, OSU03012, and OSU03013 at the indicated concentrations and cell viability was assessed 24 hours later. OSU03012 and OSU03013 killed more than 90% of the cells with 10 μmol/l of the respective inhibitors. Celecoxib at 100 μmol/l had a similar effect, but celecoxib at 50 μmol/l was not effective at reducing cell viability. This screen was conducted in replicates of four in three separate experiments. **(d) **The T47D cells similarly responded to the celecoxib analogues based on cell survival. Each experiment was performed in replicates of six on three separate occasions. **(e) **MDA-MB-453 cells were treated for 2, 4, 6, or 24 hours with Ly294002 and P-Akt was measured (top panel). A comparison was made with the MDA-MB-453 cells exposed to OSU03012 (10 μmol/l) for 2, 4, or 6 hours. Total Akt was measured as a control for sample input.

Akt plays a central role in preventing apoptosis, and therefore we investigated the potential for the celecoxib analogues to trigger programmed cell death. Subsequent to the inhibition of Akt activity, we observed a significant increase in poly(ADP-ribose) polymerase cleavage 12 and 24 hours later, indicating that the cells were undergoing apoptosis (Fig. 3a). This was consistent with nucleosomal fragmentation (Fig. 3b). We therefore concluded that the cells were undergoing apoptosis following drug treatment. The fate of the cells was followed to 24 hours, at which time cell viability was assessed. OSU03012 and OSU03013 killed the MDA-MB-453 cells in a dose dependent manner (Fig. 3c). There was a more than 90% reduction in viability with 10 μmol/l of each of the compounds. In contrast, the parent compound celecoxib had a similar effect on viability at 100 μmol/l. These studies were then extended to include the T47D breast cancer cells, and they responded in a similar manner (Fig. 3d).

It was noted that although Ly294002 inhibits P-Akt, its effect on cell viability was not as robust as that of OSU03012. To elucidate why this may be, we treated the cells with Ly294002 over periods of 2, 4, 6 and 24 hours to evaluate the possibility that it may not be stable in an aqueous solution and therefore loses its ability to inhibit P-Akt. We noted that after 6 hours the ability of Ly294002 to inhibit P-Akt began to diminish. Furthermore, by 24 hours Ly294002 only inhibited P-Akt by about 50% (Fig. 3e, top panel). In contrast, P-Akt inhibition was sustained for OSU03012 throughout the time course (Fig. 3g, bottom panel). It was not possible to measure the effect of OSU03012 after 24 hours because of the cytotoxic effect on the cells. Thus, we determined that Ly294002 was unable to sustain its inhibitory effect on P-Akt, which could explain why only 25–30% of the cells died after 24 hours.

**Figure 4:**
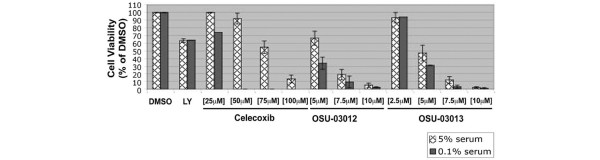
Effect of serum on the cytotoxicity of celecoxib and its analogues. MDA-MB-453 cells were treated with dimethyl sulphoxide (DMSO), Ly294002, celecoxib, OSU03012, or OSU03013 in either 5% foetal bovine serum/RPMI 1640 or 0.1% foetal bovine serum/RPMI 1640. Cell survival was measured 24 hours later using the MTT assay. Each of the treatments were tested in replicates of four and repeated twice.

The efficacy of anticancer drugs can be perturbed by problems with serum binding, and this often results in attenuated cellular effects. Therefore, we compared the cytotoxic effect of OSU03012 and OSU03013 in high (5%) verses low (0.1%) serum. This was done to ascertain whether serum had a protective effect against the celecoxib analogues. Up to this point, all of the experiments were performed in 5% foetal bovine serum/RPMI 1640 containing media, and therefore comparisons were made with cells treated in 0.1% fetal bovine serum/RPMI 1640. Serum had a remarkable protective effect against celecoxib (Fig. 4). In contrast, the serum had little effect on how well the celecoxib analogues killed the cells. These data suggest that altering the chemical structure of celecoxib not only enhanced cell killing but also increased the bioavailability of the drugs in the presence of serum.

### Frequency of phospharylated Akt expression in normal and tumor breast tissue

**Figure 5:**
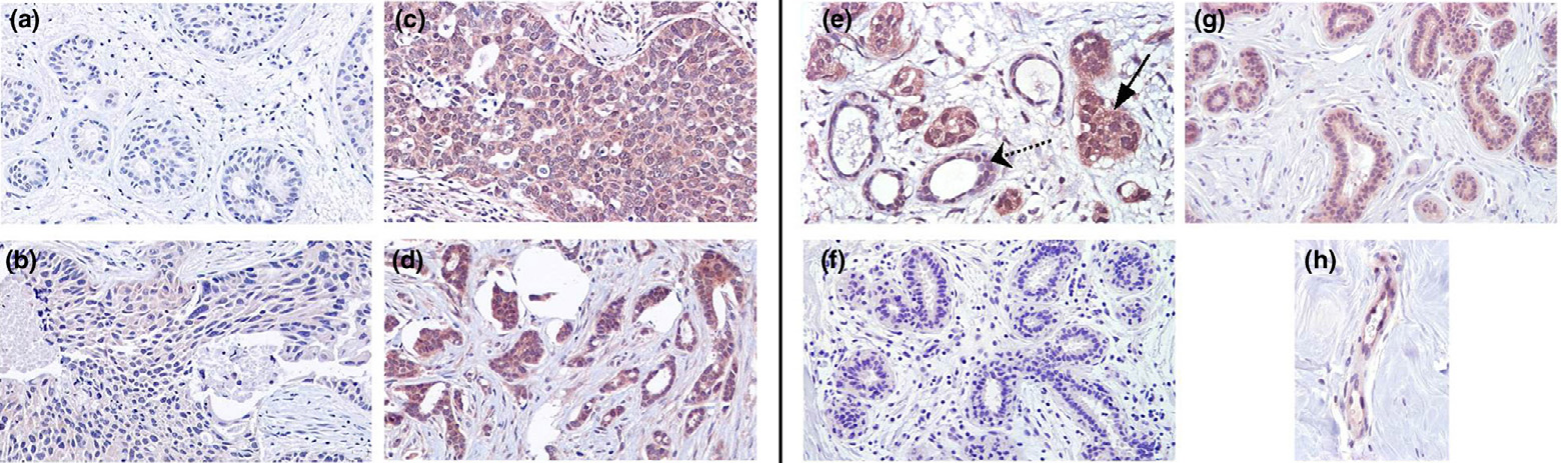
Phosphorylated Akt expression in tumour and normal tissues of the breast. **(a-d) **Phosphorylated Akt (P-Akt) staining of invasive ductal carcinomas (IDCs) of the breast ranging from undetectable to intense staining. The scoring system was from 0 to 3 and examples of such are represented in panels a-d, respectively. **(e) **Normal ducts (dashed arrow) adjacent to the IDC (solid arrow) expressed appreciably less P-Akt. **(f) **Likewise, P-Akt staining was weak when whole sections of normal breast tissue were stained. **(g) **However, some cases of normal breast tissues stained more intensely for P-Akt. **(h) **It was also noted that P-Akt was present in endothelial cells surrounding the tumours. Original magnifications: panels a-g, 200×; panel h, 400×.

In order to estimate the proportion of patients that might benefit from inhibitors to P-Akt, we screened breast tumour TMAs. A description of the patients and the clinicopathological features of their tumours are given in Additional file 1. P-Akt expression was moderately to highly expressed in 58% (221/390) of the tumours. The distribution of P-Akt expression in the 390 tumours was as follows: no staining, 43/390 (11%); weak staining, 122/390 (31%); moderate staining, 120/390 (31%); and strong staining, 105/390 (27%; Fig. 5a–d, respectively). P-Akt was predominantly expressed in epithelial cells and was noted in endothelial cells, but it was not expressed in the stroma. P-Akt was highly expressed in both oestrogen receptor positive and negative cases. There was no significant difference (*P *= 0.5839) in overall survival between patients who expressed high levels of P-Akt and those who expressed low levels of the activated protein. We also evaluated the relationship of P-Akt expression with other clinicopathologic variables, such as grade, lymph node status and histology, but we found no significant correlations (data not shown). This is most likely because we were unable to define the patient population based on treatment.

Comparisons were then made between normal and neoplastic tissues because we noted that in some instances the adjacent normal ducts expressed less P-Akt (Fig. 5e, broken arrow) compared with the tumour (Fig. 5e, solid arrow). Because the cores only represent a small amount of the tumour tissue, normal ducts were often not present. We obtained 26 normal breast tissue samples from reduction mammoplasties to examine P-Akt. In some instances, P-Akt was not expressed (Fig. 5f) whereas in others it was detectable (Fig. 5g). Overall, we determined that P-Akt was moderately to highly expressed in only 35% (9/26) of cases. We then determined that P-Akt more likely to be activated in tumours than in normal breast tissue by χ^2 ^analysis (*P *≤ 0.025). Thus, inhibitors to this pathway would hopefully affect the tumour tissue and cause few side effects in surrounding normal tissue. We also noted expression of activated Akt in endothelial cells surrounding tumours (Fig. 5h). Thus, OSU03012 and OSU03013 could potentially inhibit P-Akt in tumours as well as in the surrounding endothelial cells.

Finally, in attempt to elucidate why Akt may be activated in the tumours, we stained the tissues for HER-2 expression. Tumors that expressed high levels of HER-2 were much more likely to express activated Akt (*P *< 0.01) than were those that expressed low levels of the receptor (Table 1). Therefore, in this cohort of patients HER-2 overexpression was positively related to activated Akt, further supporting *in vitro *models of signal transduction. Together, these data show that P-Akt is frequently activated in primary breast cancers, indicating that small molecule inhibitors targeting this pathway may be useful for treating this disease.

## Discussion

In this study, we determined that OSU03012 and OSU03013 are potent inhibitors of the Akt signalling pathway, which are effective at inducing apoptosis in a breast cancer cell line that expresses high levels of HER-2. Alternatively, celecoxib was not particularly effective at inhibiting Akt or killing the MDA-MB-453 cells. Thus, the new celecoxib analogues are much better than the parent compound at inhibiting the Akt pathway. Between the two inhibitors, it appeared that OSU03012 was more specific for inhibiting the Akt pathway than was OSU03013. We also noted that the celecoxib analogues were just as effective in high serum as they were in low serum. This is in contrast to the protective effect that serum had on celecoxib. Our data is consistent with the protection afforded by serum when pancreatic cells were treated with celecoxib (50 μmol/l) [31].

Thus, it appears that structural modification of celecoxib resulted not only in increased P-Akt inhibition but also in enhanced bioavailability, at least *in vitro*. This is important to us because we move toward the development of Akt inhibitors that could be taken orally. For example, we previously reported that the oral administration of the celecoxib derivative DMC (4‑ [5-(2,5-dimethylphenyl)-3 trifluoromethyl-1H-pyrazol-1-yl]-benzene-sulfonamide) resulted in inhibition of P-Akt and ultimately suppressed development of prostate tumours [34]. This compound is structurally related to OSU03012 and OSU03013, and therefore we expect them also to be amenable to oral administration. In a recent study, OSU03012 (100 mg/kg per day) was given orally to mice bearing prostate tumours, and the intratumoural concentrations of the drug were in excess of 15 μmol/l, which coincided with tumour regression (Kulp S. personal communication). The mice tolerated the drug very well without weight loss. These data are important because our work indicates that we only need 5–10 μmol/l OSU03012 to kill highly aggressive breast cancer cells *in vitro*. Thus, if we are able to establish an intratumoural concentration in excess of 15 μmol/l, then it is very likely that this compound will have a cytotoxic effect against the MDA-MB-453 cells when studied in a xenograft model. The lack of overt toxicity is also striking and will be confirmed in models of breast cancer. Given these encouraging data, preclinical studies to this effect are currently underway in our laboratory in models of breast cancers.

We also determined that P-Akt was expressed in 58% (221/390 cases) of breast cancers. This represents one of the largest studies of P-Akt expression in breast cancer to date. The largest study reported that 49% (331/670) of breast cancer cases expressed high P-Akt [35]. Similarly, a smaller study of 40 patients reported that P-Akt was highly activated in 48% of breast cancer cases [36]. The prognostic value of P-Akt appears to depend on the types of tumours analyzed and the treatment protocol that the patients received. Like Panigrahi and coworkers [35], we did not find an association between P-Akt and patient survival. In both cases, P-Akt was examined in a large cohort of patients (about 670 cases) for whom treatment was not standardized, which could explain the lack of correlation with survival. In contrast, P-Akt was reported to be associated with poor overall survival in a subset of lymph node negative breast cancer patients (*n *= 99) for whom treatment was standardized [37]. Likewise, P-Akt predicted poor outcome among endocrine treated breast cancer patients (*n *= 93) who participated in a clinical trial using tamoxifen, goserelin, or both agents [38]. In another study [39] P-Akt was not associated with poor survival in a group of patients that was part of a controlled clinical trial examining the potential benefit of chemotherapy. The investigators found that the expression of P-Akt did not differentiate between chemotherapy responders and nonresponders. However, P-Akt was associated with a lack of response to radiotherapy. They concluded that patients were more likely to benefit from radiotherapy if their tumours were P-Akt negative.

Regarding P-Akt in normal tissues, we are the first to examine the frequency of P-Akt in normal breast tissue, in which Akt was activated in only 35% of cases, as compared with 58% of tumours. Similar to our study, basal P-Akt is low in normal ovarian surface epithelial cells compared with tumour cell lines [40]. Normal fibroblasts and colonic epithelial cells also express relatively little P-Akt compared with tumour cell lines [23]. It remains unclear what regulates P-Akt expression in normal ducts. We suspect that it relates to hormonal and/or growth factor activation of the Pi3K/Akt pathway. In particular, oestrogen and IGF-1 have been shown to stimulate the phosphorylation of Akt and induce its kinase activity in breast cancer cells [41]. Because oestrogen and IGF-1 are also present in the sera of women, it is possible that these mitogens could induce P-Akt in normal breast epithelial cells. Independent of this, we found that breast tumours consistently had higher levels of P-Akt. We estimate that breast tumours are twice as likely to express high levels of P-Akt, providing further rationale for developing inhibitors of this pathway for the treatment of cancer. Importantly, tumour cells actually depend on activated Akt for survival whereas normal cells do not [23]. This was determined using an adenoviral system that produced an Akt dominant negative inhibitor.

It could be anticipated that the celecoxib analogues might also be used in instances where resistance to standard chemotherapy has developed [42]. There are cases in which HER-2 overexpressing cells are refractory to herceptin treatment. This was illustrated in a clinical trial in which patients were recruited based on HER-2 overexpression. It was somewhat surprising that under 30% of patients responded to herceptin even though they qualified for the study based on HER-2 overexpression. Patients with amplified HER-2 had a 34% response rate whereas only 7% responded without amplification [22]. This study also pointed out that only patients with tumours that stained 3+ (*n *= 84 patients) responded to the drug whereas those staining 2+ (*n *= 27 patients) did not. The patients who did not respond were described as having tumours with moderate overexpression (2+) and/or that expressed HER-2 in the absence of gene amplification. It is noteworthy that the MDA-MB-453 cell line is a model for such tumours. They are considered to be moderate HER-2 overexpressing cells (2+) when compared with SkB-3 (3+) or BT-474 cells (3+), based on western blotting [43]. Although they overexpress HER-2, they are resistant to herceptin [43,44]. At this point, the underlying mechanism for recalcitrance to herceptin is not understood. One possible explanation for resistance is the high levels of P-Akt [43]. To examine this in more detail, constitutively activated Akt was expressed in BT-474 cells, which are known to be sensitive to Herceptin. However, expression of high P-Akt rendered the BT-474 cells insensitive to herceptin [43]. This is consistent with a report showing that that inhibiting P-Akt with Ly294002 enhances the cytotoxic effect of herceptin [17]. Inhibiting P-Akt with Ly294002 also prevents the anchorage independent growth of breast cancer cell lines that overexpress HER-2, such as the MDA-MB-453 cells [45]. A dominant negative inhibitor of the p85 subunit of Pi3K similarly blocked anchorage independent growth, providing further evidence that selective inhibition of this pathway may be particularly useful when treating HER-2 overexpressing breast cancers [45]. With respect to this, it seems reasonable that inhibiting the Akt pathway may be a way to kill breast cancer cells that have developed herceptin resistance.

Our study indicates that OSU03012 and OSU03013 inhibit P-Akt and ultimately kill herceptin resistant cells *in vitro*. This is timely, given a recent report [46] showing that the parent compound celecoxib did not benefit patients with HER-2 overexpressing tumours that were also resistant to herceptin. Thus, now more than ever there is a need to identify new agents that can be used to treat patients who have limited therapeutic options.

## Conclusion

In conclusion, celecoxib analogues provide an opportunity to inhibit P-Akt and ultimately kill breast cancer cells that overexpress HER-2.

## Abbreviations

Akt-DN = Akt dominant negative; 4EBP-1 = 4E binding protein-1; GSK = glycogen synthase kinase; HER = human epidermal growth factor receptor; IGF-1 = insulin-like growth factor-1; MAPK = mitogen-activated protein kinase; P-Akt = phosphorylated Akt; PDK = phosphoinositide-dependent kinase; PI3K = phosphatidylinositol 3-kinase; RTK = receptor tyrosine kinase; TMA = tissue microarray.

## Competing interests

The authors declare that they have no competing interests.

## Authors' contributions

JEK performed many of the western blotting experiments. She also developed the P-Akt immunostaining and assisted in writing. CL carried out some of the drug treatments and performed western blots. CC and JZ made OSU03012. CBG was the lead pathologist on the study. MC provided the biostatistical support. DH was in charge of building the tumor tissue microarray. EY performed the Her-2 immunostaining and helped optimize the P-Akt staining. JE provided the normal breast tissues, MP contributed infrastructure support. SD was the Principle investigator that designed the project, oversaw the daily activities and wrote the manuscript.

## Supplementary Material

Additional File 1 A table summarizing the characteristics of the patients and their tumours. Click here for file

## Figures and Tables

**Table 1:** Correlation between P-Akt and HER-2 proteins in primary breast tumours

	Negative (P-Akt 0,1)	Positive (P-Akt 2,3)	Total
Negative (HER2 0,1; counted/expected)	111/100.2	130/140.8	241
Positive (HER2 2,3; counted/expected)	32/42.8	71/60.2	103
Total	143	201	344

A total of 344 invasive ductal carcinomas were examined for expression of phospharylated Akt (P-Akt) and human epidermal growth factor receptor (HER)-2. A binary scoring system (0,1 versus 2,3) was used to compare staining between samples. Patients who overexpressed HER-2 were more likely to express P-Akt also. Statistical analysis was derived through the χ^2 ^test (*P *< 0.01).
