# Supplementary material for: Celecoxib analogues disrupt Akt signaling, which is commonly activated in primary breast tumours
Source: Breast Cancer Res. 2005 Aug 1;7(5):R796–807. doi: 10.1186/bcr1294 (PMC1242152; doi:10.1186/bcr1294)
Supplement: Additional File 1 — A table summarizing the characteristics of the patients and their tumours. [file bcr1294-S1.pdf]

Table 1 supplemental. Description of the patients and their tumors. The breast tumor tissue microarrays (TMA) were constructed from archival material collected between 1974-1995. Tissues for formal fixed and paraffin embedded. A total of 438 tumors were evaluated immunohistochemically for P-Akt. Each of the samples were represented on the TMA in duplicate and the concordance in scoring was >90%. Abbreviations: DCIS, ductal carcinoma in situ; IDC, infiltrating ductal carcinoma; ILC, infiltrating lobular carcinoma; NOS, not otherwise specified

Characteristics		Data
Diagnostic Age, yr		
	Mean	61.01
	Median	63.07
	Range	59.03
Lymph Node Status, n (%)		
	Negative	266 (60.7)
	Positive	126 (28.8)
	Unknown	46 (10.5)
ER status, n (%)		
	Negative	93 (21.2)
	Positive	216 (49.3)
	Unknown	129 (29.4)
Tumor Grade, n (%)		
	1	94 (21.5)
	2	236 (53.9)
	3	108 (24.6)
Tumor Size, n (%)		
	<= 5mm	4 (2.2)
	<= 1cm	64 (14.6)
	<= 2cm	142 (32.4)
	> 2cm	147 (33.6)
	Unknown	81
Histology, n (%)		
	in situ carcinoma	16 (3.6)
	IDC, NOS	353 (80.5)
	IDC, variants	24 (5.5)
	ILC	43 (10)
	IDC and ILC	2 (0.4)
Survival, yr		
	Mean	10.21
	Median	10.76
	Range	26.62
Total Follow-up, yr		
	Mean	14.47
	Median	15.4
	Range	20.34
